# Molecular Mechanisms of Resistance to Direct-Acting Antiviral (DAA) Drugs for the Treatment of Hepatitis C Virus Infections

**DOI:** 10.3390/diagnostics13193102

**Published:** 2023-09-30

**Authors:** Mohammad Asrar Izhari

**Affiliations:** Department of Laboratory Medicine, Faculty of Applied Medical Sciences, Al-Baha University, Al-Baha 65522, Saudi Arabia; aazhari@bu.edu.sa; Tel.: +966-547090882

**Keywords:** HCV, DAA, RAAS, HCC, genotype, multidrug-resistant, NS3, NS5A, NS5B

## Abstract

Hepatitis C virus (HCV) is a hepatotropic virus that affects millions of human lives worldwide. Direct-acting antiviral (DAA) regimens are the most effective HCV treatment option. However, amino acid substitution-dependent resistance to DAAs has been a major challenge. This study aimed to determine the increasing risk of DAA resistance due to substitutions in DAA target non-structural proteins (NS3/4A, NS5A, and NS5B). Using a Sequence Retrieval System (SRS) at the virus pathogen resource (ViPR/BV-BRC), *n* = 32763 target protein sequences were retrieved and analyzed for resistance-associated amino acid substitutions (RAASs) by the Sequence Feature Variant Type (SFVT) antiviral-resistance assessment modeling tool. Reference target protein sequences with 100% identity were retried from UniProt following NCBI BLAST. The types and locations of RAASs were identified and visualized by AlphaFold and PyMol. Linux-r-base/R-studio was used for the data presentation. Multi-drug-resistant variants of NS3/4A in genotype 1 (*n* = 9) and genotype 5 (*n* = 5) along with DAA-specific NS3/4A, NS5A, and NS5B variants were identified pan-genotypically. A total of 27 variants (RAASs) of all the targets were identified. Fourteen genotype 1-specific substitutions: V1196A, V1158I, D1194A/T/G, R1181K, T1080S, Q1106R, V1062A, S1148G, A1182V, Y2065N, M2000T, and L2003V were identified. The most frequent substitutions were V1062L and L2003M, followed by Q2002H. L2003V, Q2002H, M2000T, Y2065N, and NL2003M of NS5A and L2003M of NS5B conferred resistance to daclatasvir. S2702T NS5B was the sofosbuvir-resistant variant. D1194A NS3/4A was triple DAA (simeprevir, faldaprevir, and asunaprevir) resistant. The double-drug resistant variants R1181K (faldaprevir and asunaprevir), A1182V and Q1106K/R (faldaprevir and simeprevir), T1080S (faldaprevir and telaprevir), and single drug-resistant variants V1062L (telaprevir), D1194E/T (simeprevir), D1194G (asunaprevir), S1148A/G (simeprevir), and Q1106L (Boceprevir) of NS3/4A were determined. The molecular phenomenon of DAA resistance is paramount in the development of HCV drug candidates. RAASs in NS3, NS5A, and NS5B reduce the susceptibility to DAAs; therefore, continuous RAAS-dependent resistance profiling in HCV is recommended to minimize the probability of DAA therapeutic failure.

## 1. Introduction

HCV and hepatitis B virus (HBV) are the two major hepatotropic viruses [1], which are the principal etiological agents of liver cancer [2], especially hepatocellular carcinoma (HCC) [3], which continues to be a significant health concern globally [4]. Approximately > 180 million individuals are affected by HCV (a blood-born RNA virus) worldwide [5,6], with a prevalence of 1.2–1.7% of chronic HCV infection in the adult population [7]. Prolonged HCV infection and long-term damage to the hepatocytes due to combined direct and/or indirect vital oncogenic mechanisms trigger the development of HCC [8]. A recent study shows that HCV is genetically more diverse than the human immunodeficiency virus-1 [9] and produces diverse mutant clouds in infected people due to an error-prone polymerase enzyme and a high rate of mutations [9,10]. Genome-wide inquisitive analysis initially unraveled six major HCV genotypes (from HCV-1 to HCV-6) and sub-genotypes, but genotype-7 was reported later [9,11]. HCV-genotype-1 (46.2% of HCV cases) is the most prevalent genotype globally, whereas HCV-genotype-3 (30.1% of global HCV cases) is the second-most prevalent [12]. HCV-2, HCV-4, and HCV-6 contribute 22.8% of the total HCV infection burden, while genotype-5 constitutes less than 1% worldwide [12]. Genomic variation in HCV, combined with other robust mechanisms of immunological evasion, is one of the crucial factors affecting the establishment of the chronicity of the infection and the severity of the disease. HCV-induced progression and development of HCC were reported to be genotype-dependent [13,14]. Variation in the amino acid sequence of HCV strains originating from diverse genotypes and strains from different sub-types in each genotype has been estimated to be 30% and 15%, respectively [15]. The high genetic diversity of HCV poses an extraordinary challenge to developing an efficacious vaccine against HCV. However, the nonstructural (NS) proteins NS3, NS4, and NS5 are comparatively more conserved throughout the HCV genotypes than the envelope protein, making them a suitable therapeutic target [16]. Initial standard therapeutic options (a combination of an injection of pegylated interferon-α2/week with a daily oral ribavirin dose) for the treatment of HCV infection [17,18] were associated with varying degrees of adverse effects: anemia (hemolytic), fatigue, and neurological problems [19,20,21]. Successful interferon-free therapy could lead to transient cerebral atrophy, probably due to the decreased neuroinflammation and edema [22].

Apart from the side effects of the standard therapy, another challenge that impedes the effective management of HCV infection is its chronicity and potential to progress to HCC. Moreover, attaining sustained virologic response (SVR) for at least 3 or more months is a crucial parameter for measuring the effectiveness and success of the therapy [23]. The poor SRV rate (40–50%) of combination therapy ascertained the ineffectiveness of the treatment in approximately 50% of treated patients [24]. However, in 2011, two direct-acting antivirals (DAAs), namely boceprevir and telaprevir, were approved for treating HCV infections as therapeutic breakthroughs [25,26], while in 2013, a paradigm shift followed the discovery and approval of the oral use of simeprevir and sofovir [26]. These antivirals achieved up to 90 SRV rates across the HCV genotypes [26]. DDAs target HCV’s three significant nonstructural protein components: NS3/4A (with helicase and serine protease activity) [27], NS5A (three-domain protein), and NS5B (RNA-dependent RNA polymerase), vital in virus replication [28]. DAAs, either protease or polymerase inhibitors, have decreased the duration of therapy and achieved a high SRV rate [28]. The major categories of DAAs are NS3/4A inhibitors or NS3/4A protease inhibitors (PIs), NS5A inhibitors, and NS5B inhibitors (NS5B nucleotide inhibitors and nonnucleoside polymerase inhibitors) [25,29]. Though all three classes of DAAs are current therapeutic choices as they exhibit antiviral activities across the HCV genotypes and subgenotypes, studies have reported perturbation in activities of DAAs due to the emerging resistance-associated amino acid (AA) substitution (RAASs)/variants, especially at drug target sites [30,31]. Preexisting or treatment-dependent (RAASs)/variants at a particular AA position in the viral swarm could enhance the probability of the viral breakthrough and relapse of the infection [32,33,34]. Longstanding treatment-dependent RAASs, along with the substandard genetic barrier to resistance (only a few mutations may generate resistance) [35], may affect the effectiveness and/efficacy of the DAAs used for the treatment of HCV-infected people, leading to treatment discontinuation or failure [36]. Although the combination of sofovir and ledipasvir is considered as a drug of choice due to a greater degree of genetic barriers to resistance [37], however, drug-resistant variants are still critical for the established effectiveness of the DAAs. Substitutions in HCV nonstructural protein drug targets (NS3/4A, NS5A, and NS5B) remain the deciding factors in the design and development of DAAs and other antiviral agents. Though the clinical significance of the RAASs is not well demonstrated yet, their role in the development of resistance to DAAs and the reduction in efficacy of the treatment has been reported [32,34]. The current investigation aimed to determine the substitutions in these key target sites and their significant role in the emergence of RAASs/variants.

## 2. Materials and Methods

To ascertain the risk of resistance to DAAs (NS3/4A inhibitors, NS5A inhibitors, and NS5B inhibitors), the protein sequences of the drug target nonstructural proteins (NS3/4A, NS5A, and NS5B) of hepatitis C virus were retrieved from Bacterial Viral Bioinformatics Resource Center (BV-BRC)/*Virus Pathogen* Database and Analysis *Resource* (ViPR, www.viprbrc.org), USA. Multiple query datasets were generated based on the retrieved sequences to execute the risk assessment analyses. 

### 2.1. Retrieval of Target Nonstructural Protein Sequences 

Nonstructural proteins (NS3, NS5A, and NS5B) sequences were retrieved from across the HCV genotypes (from genotypes 1 to 7) by applying the in-built Sequence Retrieval System (SRS) at ViPR, now known as BV-BRC Bacterial and Viral Bioinformatics Resource Center | BV-BRC. Nonstructural protein sequences without any genotype designation (genotype unclassified/genotype UC) in the database were also retrieved, evaluated, analyzed, and included in the present study. While applying SRS, specimen attributes such as blood, plasma, serum, ascitic fluid, and cell supernatant were considered. Host attributes (across gender), virus attributes (across HCV genotypes), infection class (experimental and natural chronic), and time attributes (beyond the year 2011) were also kept under consideration while executing using SRS. 

### 2.2. Operational Definition and Query Dataset Generation

**Figure 1:**
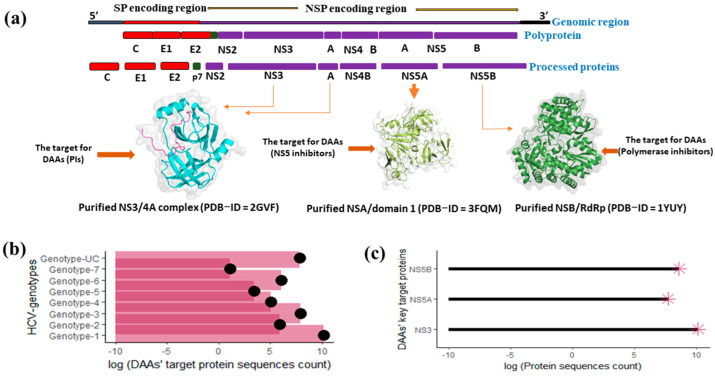
Illustration of HCV genomic regions, and DAA target protein sequence counts. (**a**) The genomic organization, polyprotein, and processed proteins, along with a 3-dimensional protein model of DAA’s target non-structural proteins: NS3, NS5A, and NS5B. (**b**) Logarithmic value of target protein sequence count by HCV genotypes. (**c**) Logarithmic value of target protein sequence count by DAA’s target protein.

RAASs in HCV polyprotein targets for DAAs may impact the efficacy of the three different categories of DAAs: NS3/4A protease inhibitors (telaprevir, asunaprevir, paritaprevir, boceprevir, and grazoprevir) with their booster protease inhibitors (ritonavir), NS5B/RdRp inhibitors (sofosbuvir), and NS5A inhibitors (daclatasvir, velpatasvir, elbasvir, ledipasvir, and ombitasvir). The Food and Drug Administration (FDA) approved various combinations of two or more antivirals from these three groups of DAAs at different times from 2011 onward for treating HCV infections caused by different genotypes [38]. As the NS3/4A, NS5A, and NS5B HCV polyproteins are the vital targets (Figure 1a) for the FDA-approved anti-HCV antivirals in use, enhanced resistance/susceptibility to FDA-approved DAAs owing to amino acid substitutions in these target proteins were investigated in the present study by employing the ViPR in-built algorithm and mathematic modeling [38,39]. I stratified the data into eight query datasets based on the genotypes: d1 = genotype 1 (*n* = 26533), d2 = genotype 2 (*n* = 371), d3 = genotype 3 (*n* = 2738), d4 = genotype 4 (*n* = 154), d5 = genotype 5 (*n* = 31), d6 = genotype 6 (*n* = 444), d7 = genotype 7 (*n* = 03), and d8 = genotype UC (*n* = 2489) before processing them (Figure 1b). Based on the types of target proteins, the data were also divided into three distinct query datasets: d1 = NS3 (*n* = 25162), d2 = NS5B (*n* = 5413), and d3 = NS5A (*n* = 2188) before being processed (Figure 1c).

### 2.3. Evaluation of RAASs in DAAs’ Target Proteins and Their Phenotypic (Resistance) Effects

**Figure 2:**
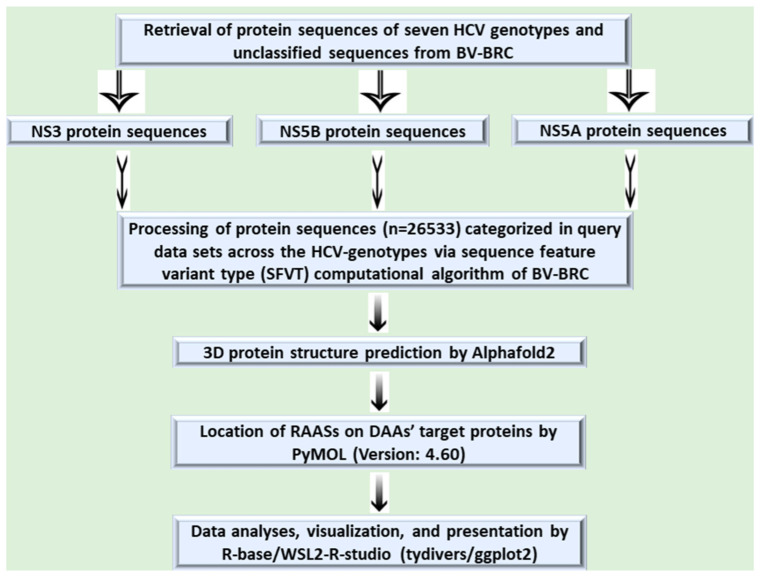
Illustration of methodology encompassing DAA’s target protein sequences, analytical resources, and tools for locating and visualizing the RAASs in NS3, NS5A, and NS5B proteins.

I determined the DAA resistance risk by employing an in-built sequence feature variant type (SFVT) of ViPR’s antiviral-resistance assessment tool, which is based on a computational algorithm. Each query dataset was purified, prepared, and separately analyzed via SFVT to gain insight into the resistance-associated amino acid substitutions (RAASs). The reference sequences for NS3 and NS5A/NS5B polyproteins used during the analyses were sequence IDs AAA72945.1 and NP_671491.1, respectively. The result was obtained following the sample run, which encompassed the enhanced resistance to any one or multiple DAAs with their allied RAASs in each query sequence of the datasets. Some RAASs without any phenotypic effect on resistance to the DAAs were excluded from the study. To access the locations of the RAASs on the respective DAA targets, each reference sequence was blasted using NCBI Protein Basic Local Alignment Search Tool BLAST: Basic Local Alignment Search Tool (nih.gov) to select NS3 and NS5A/NS5B sequences with 100% percent identity and query coverage. Based on the sequence IDs of selected sequences, I retrieved sequences from UniProt UniProt to achieve a 3D protein structure (UniProt ID for NS3:P27958 and for NS5A/NS5B: P26663). The retrieved sequences were folded for a 3D protein structure using AlphaFold2/MMSeqs2 *AlphaFold Protein Structure* Database (ebi.ac.uk). The locations of the RAASs (mutations) were ascertained and visualized by mapping the folded 3D structure of DAAs’ target proteins by employing PyMOL PyMOL | pymol.org. Data visualization and graphical context were generated by the R-package for the WSL2-based R-studio. The execution of the analytical methods has been demonstrated by a self-explanatory diagram (Figure 2).

## 3. Results 

### 3.1. Distribution of the RAASs among HCV Genotypes

**Figure 3:**
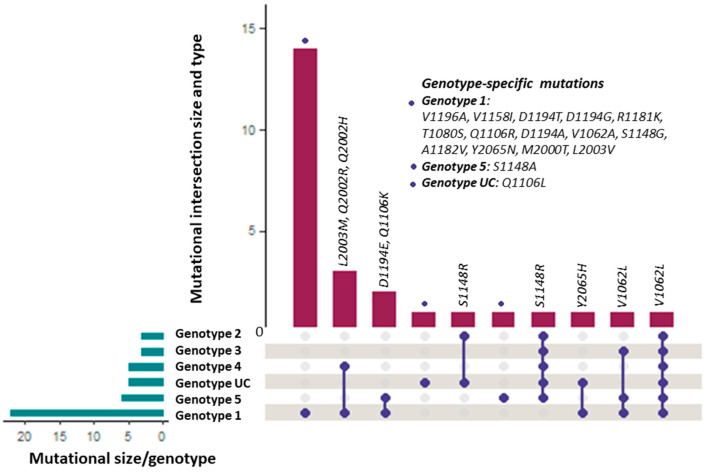
Demonstrates the presence of resistance-associated amino acid substitutions among various genotypes of the HCV in terms of intersectional size and types.

I determined the risk of anti-NS3/4A, anti-NS5A, and anti-NS5B inhibitors’ resistance across the HCV genotypes and some unclassified sequences in the form of RAASs in DAAs’ target non-structural proteins. I identified various DAAs resistance-associated NS3-mutated HCV protein sequences: twenty-three (*n* = 23) genotype 1, forty-seven (*n* = 47) genotype 2, one (*n* = 1) genotype 3, five (*n* = 5) genotype 4, twelve (*n* = 12) genotype 5, and four (*n* = 4) in genotype unclassified sequences (Table 1). The DAAs resistance-associated NS5B-mutated sequences of genotype 1, genotype 3, genotype 4, genotype 5, and unclassified genotype were four (*n* = 4), one (*n* = 1), twenty-six (*n* = 26), one (*n* = 1), and one (*n* = 1), respectively (Table 1). The DAAs resistance-associated NS5A-mutated sequences were fifty-five genotype 1 (*n* = 55), twenty-six genotype 4 (*n* = 26), and one unclassified genotype (*n* = 1), which is summarized in Table 1. Nine (*n* = 9) genotype 1 and five (*n* = 5) genotype 5 mutated NS3 protein sequences exhibited enhanced resistance (ER) to multiple DAAs (2-3 DAAs) while the rest of the mutated NS3, NS5A, and NS5B sequences were associated with resistance to a particular DAA (Table 1). RAAS V1062L was observed in genotypes 1, 2, 3, 4, 5, and unclassified (almost pan-genotypic presence). S1148R in genotypes 2, 3, 4, 5, and unclassified, Y2065H in genotype 5, unclassified, and L2003M, as well as Q2002R/H in genotypes 1, 4, and D1194E, and Q1106K in genotypes 1 and 5, were identified as shared RAAS among various HCV genotypes (Figure 3). Genotypes 1, 5, and 3 were similar because they shared one common RAASs V1062l while genotype 2 and unclassified were similar in exhibiting RAAS S1148R. Additionally, genotype-1 encompassed fourteen genotype 1-specific substitutions: V1196A, V1158I, D1194A/T/G, R1181K, T1080S, Q1106R, V1062A, S1148G, A1182V, Y2065N, M2000T, and L2003V (Figure 3). Whereas S1148S and Q1106L were identified as genotype 5- and genotype unclassified-specific resistance-associated substitutions (Figure 3). 

### 3.2. Number, Types, and Frequency of DAA Resistance-Associated Amino Acid Substitutions

**Figure 4:**
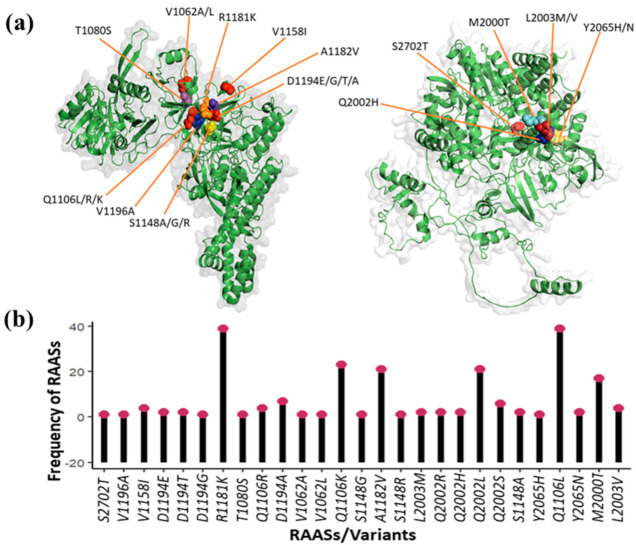
Depiction of identified key RAASs/variants of HCV. (**a**) Right side illustrates the types and location of RAASs in NS5A/NS5B, while left side shows that in NS3 HCV proteins. (**b**) Demonstrates the frequency of each RAASs/variants pan-genotypically.

The number of RAASs, their types, and their frequencies in target proteins were determined. I identified twenty-seven (*n* = 27) RAASs in total across all the DAA target proteins under consideration: S2702T, V1196A, I1158V, D1194E, D1194T, D1194G, R1181K, T1080S, Q1106R, D1194A, V1062A, V1062L, Q1106K, S1148G, A1182V, S1148R, L2003M, Q2002R, Q2002H, Q2002L, Q2002S, S1148A, Y2065H, Q1106L, Y2065N, M2000T, and L2003V (Table 1 and Figure 2b). V1062L and L2003M were observed to be highly frequent, followed by Q2002H (Figure 4b). The other three frequently identified RAASs were S1148R, Q2002R, and Y2065H (Figure 2b). Moreover, locations (at *n* = nine different positions) of unique resistance-associated substitutions in NS3 (D1194E/G/T/A, S1148A/G/R, Q1106L/R/K, V1062A/L, A1182V, V1158I, R1181K, T1080S, and V1196A), NS5A (L2003M/V, Q2002R/H, and Y2065H/N), and NS5B (S2702T, L2003M, and Q2002R/H) target proteins were spotted and detected using PyMol (Figure 2a). 

### 3.3. DAA Resistance-Associated Substitutions and Their Phenotypic Effects (Enhanced Resistance) 

I delineated various RAASs. Some of these conferred enhanced resistance to a single DAA, whereas others rendered increased resistance to multiple DDAAs (Table 1). L2003V, Q2002H, M2000T, Y2065N, and L2003M substitutions in the NS5A protein of genotype 1 led to enhanced resistance to DAA (daclatasvir), while L2003M substitution in the NS5A protein of genotypes 1 and 4 was allied with resistance to daclatasvir (Figure 4a). Moreover, Q2002H in NS5A of genotype 4 also enhanced resistance to daclatasvir. Resistance to daclatasvir was also developed by Y2065H in NS5A of genotype 1 as well as in the case of both the NS5A of genotype unclassified. S2702T in NS5B protein of genotypes 1, 3, and 5 conferred resistance against sofosbuvir (Table 1 and Figure 4a). D1194A mutations in NS3 protein foster enhanced resistance to triple DAAs: simeprevir, faldaprevir, and asunaprevir. R1181K in NS3 protein was associated with enhanced resistance to faldaprevir and asunaprevir, while A1182V and Q1106K/R in NS3 rendered increased resistance to double DAAs faldaprevir and simeprevir. T1080S in NS3 proteins was found to be linked with enhanced resistance to faldaprevir and telaprevir. Q1106K substitutions in the NS3 protein of genotype 5 were spotted to confer resistance to two DAAs (faldaprevir and simeprevir). One widespread RAAS (V1062L) in NS3 protein across genotypes 1, 2, 3, 4, 5, and unclassified developed resistance to DAA (telaprevir). However, V1062A (associated with telaprevir resistance) was only observed in genotype 1 NS3. D1194E/T (in genotypes 1 and 5 NS3) and D1194G (in genotype 1) were allied with resistance to simeprevir and asunaprevir, respectively. S1148A (genotype 5 NS3), S1148G (in genotype 1 NS3), and S1148R (in genotype 2 and unclassified NS3) were associated with DAA (simeprevir). Q1106L (in unclassified NS3 protein) developed resistance to faldaprevir. Boceprevir-associated RAAS (V1196A) was found in genotype 1 NS3.

## 4. Discussion

Despite the approval and availability of anti-NS3/4A, anti-NS5A, and anti-NS5B inhibitors for the treatment of HCV infections, naturally occurring polymorphisms (mutations) in target proteins for DAAs have been a major concern [40,41,42]. In recent years, DAAs have enhanced the sustained virologic response (SVR) rate remarkably [43]; however, due to the high replication rate and low fidelity of the HCV-RdRp enzyme, the evolution of the heterogeneous virus populations that are resistant to DAAs remains a major challenge [41]. 

In the current study, the resistance-associated amino acid substitutions in all the target proteins of DAAs, NS3 (genotypes 1, 2, 3, 4, 5, and unclassified), NS5A (genotypes 1, 4, and unclassified), and NS5B (genotypes 1, 3, 4, 5, and unclassified), have been identified, which corroborates reports in other studies [41,44,45,46,47]. RAASs fostering multiple DAAs resistance in genotypes 1 and 5 have been assessed, which is supported by the findings of Lenz et al. [48] and Jiang et al. [49]. In other genotypes, substitutions were associated with resistance to a specific DAA in genotypes 1 [48], 2 [50], 3 [50], 4 [51], 5 [49], 6 [48], and unclassified [52]. The RAASs evaluated across the HCV genotypes suggest codon-level viral non-structural protein sequence analyses that could elucidate the degree of efficacy of DAAs and the level of SVR achieved by DAAs for the treatment of HCV infection [52]. Widely reported high rates of the NS3, NS5A, and NS5B mutations that render resistance to key DAAS in clinical use could impact DAA treatment for the major HCV genotypes, and therefore, it is inferred that the impact of RAASs on treatment should be considered and assessed before the commencement of DAA treatment [42,53,54]. 

Substitution V1062L was evaluated pan-genotypically (genotypes 1, 2, 3, 4, 5, and unclassified), which is validated by the results of Keiffer et al. [51], and Lin et al. [50]. V1196A, V1158I, D1194A/T/G, R1181K, T1080S, Q1106R, V1062A, S1148G, A1182V, Y2065N, M2000T, and L2003V were found to be genotype 1-specific mutations (Figure 3), which are consistent with reports of Aguiar et al. [55], Costa et al. [56], and Dietz et al. [57]. Additionally, in this study, major NS3 variants that fostered resistance to a particular DAA and/or more than one DAA and NS5A and NS5B resistant variants to a particular DAA were determined, which suggests the possibility of treatment failure. Itakura et al. highlighted the RAASs in drug target proteins as one of the major reasons for the failure of the therapeutic management of HCV infection [58]. Lu et al. described the role of resistance-allied substitutions in non-structural protein targets for DAAs in decreasing the efficacy of the DAAs, leading to the failure of the treatment for HCV [36]. I evaluated the NS5A-resistant variants of genotype 1, L2003V, Q2002H, M2000T, Y2065N, and L2003M, leading to enhanced resistance to daclatasvir (Figure 4a), which is validated by the findings of Gao et al. [59] and Fridell et al., who demonstrated mutation-dependent enhanced resistance in both sub-types (1a and 1b) of genotype 1 [60]. In addition to that, L2003M (NS5A-resistant variant) of genotypes 1 and 4, Y2065H (NS5A-variant) of genotype 1, and Q2002H (NS5A-resistant variant) of genotype 4 were also found to be allied with resistance to daclatasvir (Figure 4a). Daclatasvir is a more potent NS5A replication inhibitor that produces a remarkable effect in decreasing viral titer [61], and it is usually given in combination with an NS5B inhibitor (sofosbuvir) [62]. Therefore, these resistant HCV variants could impact the therapeutic response of daclatasvir, leading to poor therapeutic outcomes, which is supported by the report of Costa et al., who demonstrated that NS5A mutations (in subtypes 3a and 1a) played a significant role in the poor/non-response of the combined therapy of daclatasvir and sofosbuvir [56]. 

Moreover, Stedman et al. highlighted the inhibitory effect of sofosbuvir on NS5B polymerase across the HCV genotypes [63]. S2702T (the NS5B-resistant variant) of genotypes 1, 3, and 5 conferred resistance against sofosbuvir (Table 1 and Figure 4a), which corroborated the finding of Flint et al., who reported NS5B-RAAS [64]. Furthermore, various resistant NS3 variants have been determined that confer multi-drug resistance. A triple-drug-resistant NS3 variant (D1194A) was assessed to render resistance to DAAs (simeprevir, faldaprevir, and asunaprevir), which was validated by the report on in vitro NS3/4A resistance profiling in HCV by Lenz et al. [48]. Many double-drug-resistant NS3 variants: R1181K (resistance to faldaprevir and asunaprevir), A1182V and Q1106K/R (resistance to faldaprevir and simeprevir), T1080S (resistance to faldaprevir and telaprevir), and Q1106K (resistance to faldaprevir and simeprevir) were also identified. Souman et al. reported NS3/4A protease mutation-associated asunaprevir resistance [65]. An association of a high degree of asunaprevir resistance (16 to > 280 fold) with D168A/G/H/V/Y NS3/4A variant was reported by Mc Phee et al. in HCV [66]. Mc Phee et al. also reported Q/K NS3 polymorphism [66]. Lenz et al. reported that the NS3/4A-resistant HCV genotype 1 variant D168G/N/V/I was associated with enhanced resistance to DAA (faldaprevir and simeprevir). One widespread RAAS (V1062L) in NS3 protein across genotypes 1, 2, 3, 4, 5, and unclassified developing resistance to telaprevir was identified, which corroborates with the finding of Welsch et al., who delineated the NS3/4A substitution (V36A/G/L/M and T54A/S)-dependent telaprevir resistance, which validates the finding of this study [67]. Jiang et al. explained low-grade resistance to telaprevir due to various mutations: 36C/G, R155G/I/M/S, V36A plus T54A, V36L plus R155K, T54S plus R155K, and R155T plus D168N [49]. Additionally, Jiang et al. also described high-grade resistance-associated substitutions: A156F/N/V, V36A plus R155K/T, V36M plus R155T, V36A/M plus A156T, T54A plus A156S, T54S plus A156S/T, and V36M plus T54S plus R155K, which rendered the resistance to telaprevir [49]. 

On the contrary, Wyles et al. demonstrated that there was no influence of Q to K HCV polymorphism on the treatment outcome of simeprevir and sofosbuvir in combination [68]. Boceprevir-associated RAAS (V1196A) was found in genotype 1 NS3. Tong et al. reported boceprevir resistance-associated mutations (V170A) [69]. D1194E/T and D1194G were observed to be allied with resistance to simeprevir and asunaprevir, respectively, which corroborates a description of the role of D168G/N/V/I mutations in NS3A in enhancing the resistance by Lenz et al. [48]. S1148A/G/R-dependent simeprevir resistance and Q1106L-dependent faldaprevir resistance were identified in this study, which was also explained by Lenz et al. [48]. The genetically resistant variant of HCV is genotype-dependent; therefore, the impact of RAASs should be continuously studied to ensure DAA treatment across HCV genotypes. 

## 5. Conclusions

DAAs are the most effective therapeutic options for the treatment of chronic HCV infections. However, since the DAA has been approved for clinical applications by the Food and Drug Administration (FDA), resistance-associated mutations in DAA target proteins have been the focus in order to avoid the possibility of therapeutic failure. In the present study, a wide range of amino acid substitutions in NS3A, NS5A, and NS5B target proteins for DAAs, which were associated with resistance to various FDA-approved DAAs for the treatment of HCV infection, were determined across the HCV genotypes. Multidrug-resistant NS3/4A variants in genotype 1 and genotype 5 were determined. Amino acid substitution-dependent resistance to NS3/4A protease inhibitors, NS5A, and NS5B inhibitors was assessed across the HCV genotypes. V1062L and L2003M were observed to be highly frequent, followed by Q2002H. These RAASs may impact the efficacy of the DAA-based HCV treatment, leading to virologic failure even in patients treated/retreated with multiple DAAs due to the emergence of multidrug-resistant variants of HCV. HCV-infected patients in which DAA treatment fails due to these RAASs are categorized as difficult-to-treat patients. Combination therapy SOF/VEL/VOX or SOF/GLE/PIB plus RBV for 12 weeks or without RBV for 16–24 weeks are the treatment choices for such difficult-to-treat patients. Therefore, the continuous assessment of pan-genotypic RAAS profiling in HCV is recommended to ensure the success of the DAA treatment and minimize the probability of therapeutic failure during the management of chronic HCV infection. Moreover, the execution of large-scale targeted next-generation sequencing of NS3A, NS5A, and NS5B proteins of isolated HCV genotypes/subtypes, especially from difficult-to-treat patients, could be considered for future drug design along with the current strategy of identifying in vivo and in vitro effective combinations of DAA.

## Figures and Tables

**Table 1:** Tabulation of RAASs in NS3/4A (serine protease and helicase activities), NS5A (three-domain multifunctional protein) and NS5B (RNA-dependent RNA-polymerase) DAA target proteins.

HCV Genotype-1			
QSID/RSID	RAASs/Variants	Phenotype	HCV Polyprotein
QSID-1115239302/NP_671491.1	S2702T	ER-Sofosbuvir	NS5B-polyprotein
QSID-597510766/NP_671491.1	S2702T	ER-Sofosbuvir	NS5B-polyprotein
QSID-568110881/RSID-AAA72945.1	V1196A	ER-Boceprevir	NS3-polyportein
QSID-908269165/RSID-AAA72945.1	I1158V	ER-Telaprevir	NS3-polyportein
QSID-ATY34994/RSID-AAA72945.1	D1194E	ER-Simeprevir	NS3-polyportein
QSID-ATY34994/RSID-AAA72945.1	D1194E	ER-Simeprevir	NS3-polyportein
QSID-597512356/RSID-AAA72945.1	D1194T	ER-Simeprevir	NS3-polyportein
QSID-530656976/RSID-AAA72945.1	D1194G	ER-Asunaprevir	NS3-polyportein
QSID-333611772/RSID-AAA72945.1	R1181K	ER to Faldaprevir and Asunaprevir	NS3-polyportein
QSID-568110975/RSID-AAA72945.1	T1080S	ER -Faldaprevir, ER-Telaprevir	NS3-polyportein
QSID-ATY34994/RSID-AAA72945.1	Q1106R	ER-Faldaprevir, ER-Simeprevir	NS3-polyportein
QSID-597512164/RSID-AAA72945.1	D1194A	ER-Simeprevir, ER-Faldaprevir ER-Asunaprevir	NS3-polyportein
QSID-597511348/NP_671491.1	S2702T	ER-Sofosbuvir	NS5B-polyprotein
QSID-597511368/NP_671491.1	S2702T	ER-Sofosbuvir	NS5B-polyprotein
QSID-597512292/RSID-AAA72945.1	D1194E	ER-Simeprevir	NS3-polyportein
QSID-597512292/RSID-AAA72945.1	V1062A	ER-Telaprevir	NS3-polyportein
QSID-336039226/RSID-AAA72945.1	D1194T	ER-Simeprevir	NS3-polyportein
QSID-568110888/RSID-AAA72945.1	V1062L	ER-Telaprevir	NS3-polyportein
QSID-333611678/RSID-AAA72945.1	D1194G	ER-Asunaprevir	NS3-polyportein
QSID-597512292/RSID-AAA72945.1	Q1106K	ER-Faldaprevir, ER-Simeprevir	NS3-polyportein
QSID-575502871/RSID-AAA72945.1	T1080S	ER-Faldaprevir, ER-Telaprevir	NS3-polyportein
QSID-908273241/RSID- AAA72945.1	S1148G	ES-Simeprevir	NS3-polyportein
QSID-AST22949/RSID-AAA72945.1	S1148G	ES-Simeprevir	NS3-polyportein
QSID-575502871/RSID-AAA72945.1	V1196A	ER-Boceprevir	NS3-polyportein
QSID-908268421/RSID-AAA72945.1	Q1106K	ER-Faldaprevir, ER-Simeprevir	NS3-polyportein
QSID-333611598/RSID-AAA72945.1	A1182V	ER-Faldaprevir, ER-Simeprevir	NS3-polyportein
QSID-333611772/RSID-AAA72945.1	R1181K	ER-Faldaprevir, ER-Asunaprevir	NS3-polyportein
QSID-1042527298/RSID- NP_671491.1	L2003M	ER-Daclatasvir	NS5A
QSID-1042527208/RSID-NP_671491.1	L2003M	ER-Daclatasvir	NS5A
QSID-1042527208/RSID-NP_671491.1	Q2002R	ER-Daclatasvir	NS5A
QSID-1042527168/RSID-NP_671491.1	Y2065N	ER-Daclatasvir	NS5A
QSID-1042527170/RSID-NP_671491.1	Y2065N	ER-Daclatasvir	NS5A
QSID-1042527258/RSID-NP_671491.1	L2003M	ER-Daclatasvir	NS5A
QSID-1042527258/RSID-NP_671491.1	Q2002R	ER-Daclatasvir	NS5A
QSID-1042527264/RSID-NP_671491.1	L2003M	ER-Daclatasvir	NS5A
QSID-1042527264/RSID-NP_671491.1	Q2002R	ER-Daclatasvir	NS5A
QSID-1042527266/RSID-NP_671491.1	L2003M	ER-Daclatasvir	NS5A
QSID-1042527266/RSID-NP_671491.1	Q2002R	ER-Daclatasvir	NS5A
QSID-1042527262/RSID-NP_671491.1	L2003M	ER-Daclatasvir	NS5A
QSID-1042527262/RSID-NP_671491.1	Q2002R	ER-Daclatasvir	NS5A
QSID-1042527268/RSID-NP_671491.1	L2003M	ER-Daclatasvir	NS5A
QSID-1042527260/RSID-NP_671491.1	L2003M	ER-Daclatasvir	NS5A
QSID-1042527488/RSID-NP_671491.1	Q2002H	ER-Daclatasvir	NS5A
QSID-1115239468/RSID-NP_671491.1	L2003M	ER-Daclatasvir	NS5A
QSID-1042527490/RSID-NP_671491.1	Q2002H	ER-Daclatasvir	NS5A
QSID-1042527492/RSID-NP_671491.1	Q2002H	ER-Daclatasvir	NS5A
QSID-1042527486/RSID-NP_671491.1	Q2002H	ER-Daclatasvir	NS5A
QSID-1115239512/RSID-NP_671491.1	M2000T	ER-Daclatasvir	NS5A
QSID-1115239510/RSID-NP_671491.1	M2000T	ER-Daclatasvir	NS5A
QSID-1042527484/RSID-NP_671491.1	Q2002H	ER-Daclatasvir	NS5A
QSID-1042527498/RSID-NP_671491.1	Q2002H	ER-Daclatasvir	NS5A
QSID-1042527494/RSID-NP_671491.1	Q2002H	ER-Daclatasvir	NS5A
QSID-1115239524/RSID-NP_671491.1	Y2065H	ER-Daclatasvir	NS5A
QSID-1042527496/RSID-NP_671491.1	Q2002H	ER-Daclatasvir	NS5A
QSID-1153219284/RSID-NP_671491.1	M2000T	ER-Daclatasvir	NS5A
QSID-1115239648/RSID-NP_671491.1	Y2065H	ER-Daclatasvir	NS5A
QSID-1115239656/RSID-NP_671491.1	M2000T	ER-Daclatasvir	NS5A
QSID-1115239656/RSID-NP_671491.1	Q2002R	ER-Daclatasvir	NS5A
QSID-1115239608/RSID-NP_671491.1	Y2065H	ER-Daclatasvir	NS5A
QSID-1115239608/RSID-NP_671491.1	Q2002H	ER-Daclatasvir	NS5A
QSID-1115239606/RSID-NP_671491.1	Y2065H	ER-Daclatasvir	NS5A
QSID-1115239606/RSID-NP_671491.1	Q2002H	ER-Daclatasvir	NS5A
QSID-1115239600/RSID-NP_671491.1	Y2065N	ER-Daclatasvir	NS5A
QSID-1115239620/RSID-NP_671491.1	Y2065H	ER-Daclatasvir	NS5A
QSID-1115239564/RSID-NP_671491.1	Q2002R	ER-Daclatasvir	NS5A
QSID-1115239582/RSID-NP_671491.1	L2003M	ER-Daclatasvir	NS5A
QSID-1115239584/RSID-NP_671491.1	L2003M	ER-Daclatasvir	NS5A
QSID-1115239590/RSID-NP_671491.1	Q2002H	ER-Daclatasvir	NS5A
QSID-1115239592/RSID-NP_671491.1	Q2002H	ER-Daclatasvir	NS5A
QSID-1042527576/RSID-NP_671491.1	L2003M	ER-Daclatasvir	NS5A
QSID-1042527186/RSID-NP_671491.1	Y2065H	ER-Daclatasvir	NS5A
QSID-1042527180/RSID-NP_671491.1	Y2065H	ER-Daclatasvir	NS5A
QSID-1042527188/RSID-NP_671491.1	Y2065H	ER-Daclatasvir	NS5A
QSID-1042527182/RSID-NP_671491.1	Y2065H	ER-Daclatasvir	NS5A
QSID-1115239556/RSID-NP_671491.1	L2003M	ER-Daclatasvir	NS5A
QSID-1042527192/RSID-NP_671491.1	Y2065H	ER-Daclatasvir	NS5A
QSID-1042527178/RSID-NP_671491.1	Y2065H	ER-Daclatasvir	NS5A
QSID-1042527184/RSID-NP_671491.1	Y2065H	ER-Daclatasvir	NS5A
QSID-1042527190/RSID-NP_671491.1	Y2065H	ER-Daclatasvir	NS5A
QSID-1042527608/RSID-NP_671491.1	L2003V	ER-Daclatasvir	NS5A
QSID-808181800/RSID-NP_671491.1	Y2065N	ER-Daclatasvir	NS5A
QSID-808181816/RSID-NP_671491.1	Q2002H	ER-Daclatasvir	NS5A
HCV Genotype 2			
QSID/RSID	RAASs/variants	Phenotype	HCV polyprotein
QSID-1152728359/RSID-AAA72945.1	V1062L	ER-Telaprevir	NS3-polyportein
QSID-1152728359/RSID-AAA72945.1	S1148R	ER-Simeprevir	NS3-polyportein
QSID-544168876/RSID-AAA72945.1	V1062L	ER-Telaprevir	NS3-polyportein
QSID-544168876/RSID-AAA72945.1	S1148R	ER-Simeprevir	NS3-polyportein
QSID-401712474/RSID-AAA72945.1	V1062L	ER-Telaprevir	NS3-polyportein
QSID-401712474/RSID-AAA72945.1	S1148R	ER-Simeprevir	NS3-polyportein
QSID-1152728369/RSID-AAA72945.1	V1062L	ER-Telaprevir	NS3-polyportein
QSID-1152728369/RSID-AAA72945.1	S1148R	ER-Simeprevir	NS3-polyportein
QSID-ATY35029/RSID-AAA72945.1	V1062L	ER-Telaprevir	NS3-polyportein
QSID-ATY35029/RSID-AAA72945.1	S1148R	ER-Simeprevir	NS3-polyportein
QSID-401712520/RSID-AAA72945.1	V1062L	ER-Telaprevir	NS3-polyportein
QSID-401712520/RSID-AAA72945.1	S1148R	ER-Simeprevir	NS3-polyportein
QSID-1152728313/RSID-AAA72945.1	V1062L	ER-Telaprevir	NS3-polyportein
QSID-509263121/RSID-AAA72945.1	V1062L	ER-Telaprevir	NS3-polyportein
QSID-1152728311/RSID-AAA72945.1	V1062L	ER-Telaprevir	NS3-polyportein
QSID-ATY35005/RSID-AAA72945.1	V1062L	ER-Telaprevir	NS3-polyportein
QSID-ATY35005/RSID-AAA72945.1	S1148R	ER-Simeprevir	NS3-polyportein
QSID-1152728355/RSID-AAA72945.1	V1062L	ER-Telaprevir	NS3-polyportein
QSID-1152728355/RSID-AAA72945.1	S1148R	ER-Simeprevir	NS3-polyportein
QSID-1152728371/RSID-AAA72945.1	V1062L	ER-Telaprevir	NS3-polyportein
QSID-1152728371/RSID-AAA72945.1	S1148R	ER-Simeprevir	NS3-polyportein
QSID-ATY35003/RSID-AAA72945.1	V1062L	ER-Telaprevir	NS3-polyportein
QSID-ATY35003/RSID-AAA72945.1	S1148R	ER-Simeprevir	NS3-polyportein
QSID-1152728391/RSID-AAA72945.1	V1062L	ER-Telaprevir	NS3-polyportein
QSID-1152728391/RSID-AAA72945.1	S1148R	ER-Simeprevir	NS3-polyportein
QSID-544168878/RSID-AAA72945.1	V1062L	ER-Telaprevir	NS3-polyportein
QSID-544168878/RSID-AAA72945.1	S1148R	ER-Simeprevir	NS3-polyportein
QSID-ATY35030/RSID-AAA72945.1	V1062L	ER-Telaprevir	NS3-polyportein
QSID-ATY35030/RSID-AAA72945.1	S1148R	ER-Simeprevir	NS3-polyportein
QSID-544168870/RSID-AAA72945.1	V1062L	ER-Telaprevir	NS3-polyportein
QSID-544168870/RSID-AAA72945.1	S1148R	ER-Simeprevir	NS3-polyportein
QSID-1152728397/RSID-AAA72945.1	V1062L	ER-Telaprevir	NS3-polyportein
QSID-1152728397/RSID-AAA72945.1	S1148R	ER-Simeprevir	NS3-polyportein
QSID-1152728395/RSID-AAA72945.1	S1148R	ER-Simeprevir	NS3-polyportein
QSID-1152728379/RSID-AAA72945.1	V1062L	ER-Telaprevir	NS3-polyportein
QSID-1152728379/RSID-AAA72945.1	S1148R	ER-Simeprevir	NS3-polyportein
QSID-576294944/RSID-AAA72945.1	V1062L	ER-Telaprevir	NS3-polyportein
QSID-393714879/RSID-AAA72945.1	V1062L	ER-Telaprevir	NS3-polyportein
QSID-401712478/RSID-AAA72945.1	V1062L	ER-Telaprevir	NS3-polyportein
QSID-401712478/RSID-AAA72945.1	S1148R	ER-Simeprevir	NS3-polyportein
QSID-544168874/RSID-AAA72945.1	V1062L	ER-Telaprevir	NS3-polyportein
QSID-544168874/RSID-AAA72945.1	S1148R	ER-Simeprevir	NS3-polyportein
QSID-1152728331/RSID-AAA72945.1	V1062L	ER-Telaprevir	NS3-polyportein
QSID-ATY35031/RSID-AAA72945.1	V1062L	ER-Telaprevir	NS3-polyportein
QSID-ATY35031/RSID-AAA72945.1	S1148R	ER-Simeprevir	NS3-polyportein
QSID-401712476/RSID-AAA72945.1	V1062L	ER-Telaprevir	NS3-polyportein
QSID-401712476/RSID-AAA72945.1	S1148R	ER-Simeprevir	NS3-polyportein
HCV Genotype 3			
QSID/RSID	RAASs/variants	Phenotype	HCV polyprotein
QSID-ART89572/NP_671491.1	S2702T	ER-Sofosbuvir	NS5B-polyprotein
QSID-1152728501/RSID-AAA72945.1	V1062L	ER-Telaprevir	NS3-polyportein
HCV Genotype 4			
QSID/RSID	RAASs/variants	Phenotype	HCV polyprotein
QSID-475628336/RSID-AAA72945.1	V1062L	ER-Telaprevir	NS3-polyportein
QSID-475628354/RSID-AAA72945.1	V1062L	ER-Telaprevir	NS3-polyportein
QSID-751660972/NP_671491.1	L2003M	ER-Daclatasvir	NS5A
QSID-751660974/NP_671491.1	L2003M	ER-Daclatasvir	NS5A
QSID-751660974/NP_671491.1	Q2002R	ER-Daclatasvir	NS5A
QSID-ATY35065/NP_671491.1	L2003M	ER-Daclatasvir	NS5A
QSID-ATY35065/NP_671491.1	Q2002R	ER-Daclatasvir	NS5A
QSID-ARR74221/RSID-AAA72945.1	V1062L	ER-Telaprevir	NS3-polyportein
QSID-ATY35035/RSID-AAA72945.1	V1062L	ER-Telaprevir	NS3-polyportein
QSID-ATY35066/NP_671491.1	L2003M	ER-Daclatasvir	NS5A
QSID-ATY35066/NP_671491.1	Q2002R	ER-Daclatasvir	NS5A
QSID-1042527988/NP_671491.1	L2003M	ER-Daclatasvir	NS5A
QSID-1042527996/NP_671491.1	Q2002H	ER-Daclatasvir	NS5A
QSID-1042527990/NP_671491.1	L2003M	ER-Daclatasvir	NS5A
QSID-1042527990/NP_671491.1	Q2002H	ER-Daclatasvir	NS5A
QSID-1042527998/NP_671491.1	Q2002H	ER-Daclatasvir	NS5A
QSID-1042528000/NP_671491.1	Q2002H	ER-Daclatasvir	NS5A
QSID-1042527992/NP_671491.1	L2003M	ER-Daclatasvir	NS5A
QSID-1042527992/NP_671491.1	Q2002H	ER-Daclatasvir	NS5A
QSID-1042527994/NP_671491.1	L2003M	ER-Daclatasvir	NS5A
QSID-1042527994/NP_671491.1	Q2002H	ER-Daclatasvir	NS5A
QSID-1009028115/RSID-AAA72945.1	V1062L	ER-Telaprevir	NS5A
QSID-1009028115/NP_671491.1	L2003M	ER-Daclatasvir	NS5A
QSID-1009028115/NP_671491.1	Q2002R	ER-Daclatasvir	NS5A
QSID-1009028127/NP_671491.1	L2003M	ER-Daclatasvir	NS5A
QSID-1009028113/NP_671491.1	L2003M	ER-Daclatasvir	NS5A
QSID-1009028113/NP_671491.1	Q2002R	ER-Daclatasvir	NS5A
QSID-1009028111/NP_671491.1	L2003M	ER-Daclatasvir	NS5A
QSID-1009028111/NP_671491.1	Q2002R	ER-Daclatasvir	NS5A
QSID-ATY35085/NP_671491.1	L2003M	ER-Daclatasvir	NS5A
QSID-ATY35085/NP_671491.1	Q2002R	ER-Daclatasvir	NS5A
QSID-1009028115/RSID- NP_671491.1	L2003M	ER-Daclatasvir	NS5A
QSID-1009028115/RSID-NP_671491.1	Q2002R	ER-Daclatasvir	NS5A
QSID-1009028127/RSID-NP_671491.1	L2003M	ER-Daclatasvir	NS5A
QSID-1009028127/RSID-NP_671491.1	L2003M	ER-Daclatasvir	NS5A
QSID-1009028113/RSID-NP_671491.1	Q2002R	ER-Daclatasvir	NS5A
QSID-1009028111/RSID-NP_671491.1	L2003M	ER-Daclatasvir	NS5A
QSID-1009028111/RSID-NP_671491.1	Q2002R	ER-Daclatasvir	NS5A
QSID-ATY35085/RSID-NP_671491.1	L2003M	ER-Daclatasvir	NS5A
QSID-ATY35085/RSID-NP_671491.1	Q2002R	ER-Daclatasvir	NS5A
QSID-751660972/RSID-NP_671491.1	L2003M	ER-Daclatasvir	NS5A
QSID-751660974/RSID-NP_671491.1	L2003M	ER-Daclatasvir	NS5A
QSID-751660974/RSID-NP_671491.1	Q2002R	ER-Daclatasvir	NS5A
QSID-ATY35065/RSID-NP_671491.1	L2003M	ER-Daclatasvir	NS5A
QSID-ATY35065/RSID-NP_671491.1	Q2002R	ER-Daclatasvir	NS5A
QSID-ATY35066/RSID-NP_671491.1	L2003M	ER-Daclatasvir	NS5A
QSID-ATY35066/RSID-NP_671491.1	Q2002R	ER-Daclatasvir	NS5A
QSID-1042527988/RSID-NP_671491.1	L2003M	ER-Daclatasvir	NS5A
QSID-1042527996/RSID-NP_671491.1	Q2002H	ER-Daclatasvir	NS5A
QSID-1042527990/RSID-NP_671491.1	L2003M	ER-Daclatasvir	NS5A
QSID-1042527990/RSID-NP_671491.1	Q2002H	ER-Daclatasvir	NS5A
QSID-1042527998/RSID-NP_671491.1	Q2002H	ER-Daclatasvir	NS5A
QSID-1042528000/RSID-NP_671491.1	Q2002H	ER-Daclatasvir	NS5A
QSID-1042527992/RSID-NP_671491.1	L2003M	ER-Daclatasvir	NS5A
QSID-1042527992/RSID-NP_671491.1	Q2002H	ER-Daclatasvir	NS5A
QSID-1042527994/RSID-NP_671491.1	L2003M	ER-Daclatasvir	NS5A
QSID-1042527994/RSID-NP_671491.1	Q2002H	ER-Daclatasvir	NS5A
HCV Genotype 5			
QSID/RSID	RAASs/variants	Phenotype	HCV polyprotein
QSID-751660976/RSID-AAA72945.1	Q1106K	ER-Faldaprevir, ER-Simeprevir	NS3-polyportein
QSID-ATA65699/NP_671491.1	S2702T	ER-Sofosbuvir	NS5B-polyprotein
QSID-1026671962/RSID-AAA72945.1	V1062L	ER-Telaprevir	NS3-polyportein
QSID-1026671962/RSID-AAA72945.1	D1194E	ER-Simeprevir	NS3-polyportein
QSID-751660976/RSID-AAA72945.1	V1062L	ER-Telaprevir	NS3-polyportein
QSID-1026671962/RSID-AAA72945.1	Q1106K	ER-Faldaprevir, ER-Simeprevir	NS3-polyportein
QSID-1026671954/RSID-AAA72945.1	V1062L	ER-Telaprevir	NS3-polyportein
QSID-1009028101/RSID-AAA72945.1	V1062L	ER-Telaprevir	NS3-polyportein
QSID-1009028101/RSID-AAA72945.1	S1148A	ES-Simeprevir	NS3-polyportein
QSID-1009028103/RSID-AAA72945.1	V1062L	ER-Telaprevir	NS3-polyportein
QSID-1009028103/RSID-AAA72945.1	S1148A	ES-Simeprevir	NS3-polyportein
QSID-1009028103/RSID-AAA72945.1	Q1106K	ER-Faldaprevir, ER-Simeprevir	NS3-polyportein
QSID-1026671954/RSID-AAA72945.1	Q1106K	ER-Faldaprevir, ER-Simeprevir	NS3-polyportein
QSID-1009028101/RSID-AAA72945.1	Q1106K	ER-Faldaprevir, ER-Simeprevir	NS3-polyportein
HCV Genotype UC			
QSID/RSID	RAASs/variants	Phenotype	HCV polyprotein
QSID-ART89485/NP_671491.1	Y2065H	ER-Daclatasvir	NS5A
QSID-ARB18146/RSID-AAA72945.1	V1062L	ER-Telaprevir	NS3-polyportein
QSID-844573227/RSID-AAA72945.1	Q1106L	ER-Faldaprevir	NS3-polyportein
QSID-ASE05938/RSID-AAA72945.1	S1148R	ER-Simeprevir	NS3-polyportein
QSID-ASE05936/RSID-AAA72945.1	V1062L	ER-Telaprevir	NS3-polyportein
QSID-ART89485/RSID- NP_671491.1	Y2065H	ER-Daclatasvir	NS5A

ER = enhanced resistance, QSID = query sequence identification (ID), RSID = reference sequence identification (ID), UC = unclassified HCV sequence, and RAASs = resistance-associated amino acid substitutions.

## Data Availability

The data supporting this investigation are available from the corresponding author and can be shared based on necessities.

## References

[1] Kiyasu P.K., Caldwell S.H. (1993). Diagnosis and treatment of the major hepatotropic viruses. Am. J. Med. Sci..

[2] Craig A.J., Von Felden J., Garcia-Lezana T., Sarcognato S., Villanueva A. (2020). Tumour evolution in hepatocellular carcinoma. Nat. Rev. Gastroenterol. Hepatol..

[3] Cougot D., Neuveut C., Buendia M.A. (2005). HBV induced carcinogenesis. J. Clin. Virol..

[4] Llovet J.M., Kelley R.K., Villanueva A., Singal A.G., Pikarsky E., Roayaie S., Lencioni R., Koike K., Zucman-Rossi J., Finn R.S. (2021). Hepatocellular carcinoma. Nat. Rev. Dis. Primers.

[5] Petruzziello A., Marigliano S., Loquercio G., Cozzolino A., Cacciapuoti C. (2016). Global epidemiology of hepatitis C virus infection: An up-date of the distribution and circulation of hepatitis C virus genotypes. World J. Gastroenterol..

[6] Ali A., Zaman B., Shoaib M.A., Dhanani R., Khan R. (2013). CAUSES OF PREVALENCE OF HEPATITIS–C IN VILLAGE MALKANI SHARIF, DISTRICT BADIN, SINDH, PAKISTAN. FUUAST J. Biol..

[7] Gower E., Estes C., Blach S., Razavi-Shearer K., Razavi H. (2014). Global epidemiology and genotype distribution of the hepatitis C virus infection. J. Hepatol..

[8] Goto K., Roca Suarez A.A., Wrensch F., Baumert T.F., Lupberger J. (2020). Hepatitis C Virus and Hepatocellular Carcinoma: When the Host Loses Its Grip. Int. J. Mol. Sci..

[9] Bailey J.R., Barnes E., Cox A.L. (2019). Approaches, Progress, and Challenges to Hepatitis C Vaccine Development. Gastroenterology.

[10] Domingo E., Perales C. (2019). Viral quasispecies. PLoS Genet..

[11] Murphy D.G., Sablon E., Chamberland J., Fournier E., Dandavino R., Tremblay C.L. (2015). Hepatitis C virus genotype 7, a new genotype originating from central Africa. J. Clin. Microbiol..

[12] Messina J.P., Humphreys I., Flaxman A., Brown A., Cooke G.S., Pybus O.G., Barnes E. (2015). Global distribution and prevalence of hepatitis C virus genotypes. Hepatology.

[13] Bruno S., Crosignani A., Maisonneuve P., Rossi S., Silini E., Mondelli M.U. (2007). Hepatitis C virus genotype 1b as a major risk factor associated with hepatocellular carcinoma in patients with cirrhosis: A seventeen-year prospective cohort study. Hepatology.

[14] Vescovo T., Refolo G., Vitagliano G., Fimia G.M., Piacentini M. (2016). Molecular mechanisms of hepatitis C virus–induced hepatocellular carcinoma. Clin. Microbiol. Infect..

[15] Bukh J. (2016). The history of hepatitis C virus (HCV): Basic research reveals unique features in phylogeny, evolution and the viral life cycle with new perspectives for epidemic control. J. Hepatol..

[16] Ward S., Lauer G., Isba R., Walker B., Klenerman P. (2002). Cellular immune responses against hepatitis C virus: The evidence base 2002. Clin. Exp. Immunol..

[17] Manns M.P., McHutchison J.G., Gordon S.C., Rustgi V.K., Shiffman M., Reindollar R., Goodman Z.D., Koury K., Ling M.-H., Albrecht J.K. (2001). Peginterferon alfa-2b plus ribavirin compared with interferon alfa-2b plus ribavirin for initial treatment of chronic hepatitis C: A randomised trial. Lancet.

[18] Strader D.B., Wright T., Thomas D.L., Seeff L.B. (2004). Diagnosis, management, and treatment of hepatitis C. Hepatology.

[19] Lebray P., Nalpas B., Vallet-Pichard A., Broissand C., Sobesky R., Serpaggi J., Fontaine H., Pol S. (2005). The impact of haematopoietic growth factors on the management and efficacy of antiviral treatment in patients with hepatitis C virus. Antivir. Ther..

[20] Schaefer M., Schmidt F., Folwaczny C., Lorenz R., Martin G., Schindlbeck N., Heldwein W., Soyka M., Grunze H., Koenig A. (2003). Adherence and mental side effects during hepatitis C treatment with interferon alfa and ribavirin in psychiatric risk groups. Hepatology.

[21] Toniutto P., Fabris C., Fumo E., Apollonio L., Caldato M., Avellini C., Minisini R., Pirisi M. (2005). Pegylated versus standard interferon-α in antiviral regimens for post-transplant recurrent hepatitis C: Comparison of tolerability and efficacy. J. Gastroenterol. Hepatol..

[22] Marciniewicz E., Podgórski P., Pawłowski T., Małyszczak K., Fleischer-Stępniewska K., Knysz B., Waliszewska-Prosół M., Żelwetro A., Rymer W., Inglot M. (2019). Evaluation of brain volume alterations in HCV-infected patients after interferon-free therapy: A pilot study. J. Neurol. Sci..

[23] Araújo A.R., Peruhype-Magalhães V., Coelho-dos-Reis J.G.A., Chaves L.P.V., de Lima T.A., Pimentel J.P.D., de Paula L., de Almeida C.M., Tarragô A.M., Tateno A. (2013). Dual role of IL-12 in the therapeutic efficacy or failure during combined PEG-Interferon-α2A and ribavirin therapy in patients with chronic hepatitis C. Immunol. Lett..

[24] Hadziyannis S.J., Sette H., Morgan T.R., Balan V., Diago M., Marcellin P., Ramadori G., Bodenheimer Jr H., Bernstein D., Rizzetto M. (2004). Peginterferon-α2a and ribavirin combination therapy in chronic hepatitis C: A randomized study of treatment duration and ribavirin dose. Ann. Intern. Med..

[25] Asselah T., Boyer N., Saadoun D., Martinot-Peignoux M., Marcellin P. (2016). Direct-acting antivirals for the treatment of hepatitis C virus infection: Optimizing current IFN-free treatment and future perspectives. Liver Int..

[26] Kish T., Aziz A., Sorio M. (2017). Hepatitis C in a New Era: A Review of Current Therapies. Pharm. Ther..

[27] Courcambeck J., Bouzidi M., Perbost R., Jouirou B., Amrani N., Cacoub P., Pèpe G., Sabatier J.-M., Halfon P. (2006). Resistance of hepatitis C virus to NS3–4A protease inhibitors: Mechanisms of drug resistance induced by R155Q, A156T, D168A and D168V mutations. Antivir. Ther..

[28] Lok A.S.-F. (2013). HCV NS5A inhibitors in development. Clin. Liver Dis..

[29] Herbst D.A., Reddy K.R. (2013). NS5A inhibitor, daclatasvir, for the treatment of chronic hepatitis C virus infection. Expert. Opin. Investig. Drugs.

[30] Ng T.I., Tripathi R., Reisch T., Lu L., Middleton T., Hopkins T.A., Pithawalla R., Irvin M., Dekhtyar T., Krishnan P. (2018). In vitro antiviral activity and resistance profile of the next-generation hepatitis C virus NS3/4A protease inhibitor glecaprevir. Antimicrob. Agents Chemother..

[31] Pawlotsky J.-M. (2016). Hepatitis C virus resistance to direct-acting antiviral drugs in interferon-free regimens. Gastroenterology.

[32] Harrington P.R., Komatsu T.E., Deming D.J., Donaldson E.F., O’Rear J.J., Naeger L.K. (2018). Impact of hepatitis C virus polymorphisms on direct-acting antiviral treatment efficacy: Regulatory analyses and perspectives. Hepatology.

[33] Sarrazin C. (2016). The importance of resistance to direct antiviral drugs in HCV infection in clinical practice. J. Hepatol..

[34] Shahid I., Ibrahim M.M., Nawaz M.U., Imam M.T., AlMalki W.H. (2018). Resistance-Associated Substitutions/Variants Correlate to Therapeutic Outcomes of Novel Direct-Acting Antivirals in Different HCV Genotype Treated Individuals.

[35] Bull R.A., Luciani F., McElroy K., Gaudieri S., Pham S.T., Chopra A., Cameron B., Maher L., Dore G.J., White P.A. (2011). Sequential bottlenecks drive viral evolution in early acute hepatitis C virus infection. PLoS Pathog..

[36] Lu J., Feng Y., Chen L., Zeng Z., Liu X., Cai W., Wang H., Guo X., Zhou H., Tao W. (2019). Subtype-specific prevalence of hepatitis C virus NS5A resistance associated substitutions in Mainland China. Front. Microbiol..

[37] Gritsenko D., Hughes G. (2015). Ledipasvir/Sofosbuvir (harvoni): Improving options for hepatitis C virus infection. Pharm. Ther..

[38] Geddawy A., Ibrahim Y.F., Elbahie N.M., Ibrahim M.A. (2017). Direct Acting Anti-hepatitis C Virus Drugs: Clinical Pharmacology and Future Direction. J. Transl. Int. Med..

[39] Ghany M.G., Nelson D.R., Strader D.B., Thomas D.L., Seeff L.B. (2011). An update on treatment of genotype 1 chronic hepatitis C virus infection: 2011 practice guideline by the American Association for the Study of Liver Diseases. Hepatology.

[40] Martínez A.P., Culasso A.C., Pérez P.S., Romano V., Campos R.H., Ridruejo E., García G., Di Lello F.A. (2017). Polymorphisms associated with resistance to protease inhibitors in naïve patients infected with hepatitis C virus genotype 1 in Argentina: Low prevalence of Q80K. Virus Res..

[41] Sarrazin C., Dvory-Sobol H., Svarovskaia E.S., Doehle B.P., Pang P.S., Chuang S.-M., Ma J., Ding X., Afdhal N.H., Kowdley K.V. (2016). Prevalence of resistance-associated substitutions in HCV NS5A, NS5B, or NS3 and outcomes of treatment with ledipasvir and sofosbuvir. Gastroenterology.

[42] Paolucci S., Fiorina L., Mariani B., Gulminetti R., Novati S., Barbarini G., Bruno R., Baldanti F. (2013). Naturally occurring resistance mutations to inhibitors of HCV NS5A region and NS5B polymerase in DAA treatment-naive patients. Virol. J..

[43] Liang T.J., Ghany M.G. (2013). Current and future therapies for hepatitis C virus infection. N. Engl. J. Med..

[44] Nejabat N., Hosseini S.Y., Sarvari J., Gorzin A.A., Fattahi M.R., Rasoolian M. (2019). The Investigation of Drug Resistance Substitutions in NS3 Protease Sequence of Hepatitis C Virus from Non-Responder Patients. Asian Pac. J. Cancer Prev. APJCP.

[45] Zeuzem S., Mizokami M., Pianko S., Mangia A., Han K.-H., Martin R., Svarovskaia E., Dvory-Sobol H., Doehle B., Hedskog C. (2017). NS5A resistance-associated substitutions in patients with genotype 1 hepatitis C virus: Prevalence and effect on treatment outcome. J. Hepatol..

[46] Bertoli A., Sorbo M.C., Aragri M., Lenci I., Teti E., Polilli E., Di Maio V.C., Gianserra L., Biliotti E., Masetti C. (2018). Prevalence of single and multiple natural NS3, NS5A and NS5B resistance-associated substitutions in hepatitis C virus genotypes 1–4 in Italy. Sci. Rep..

[47] Wei L., Omata M., Lim Y.-S., Xie Q., Hou J.L., Jia J., Hedskog C., Martin R., Doehle B., Yang J. (2018). HCV phylogenetic signature and prevalence of pretreatment NS5A and NS5B NI-Resistance associated substitutions in HCV-Infected patients in Mainland China. Antivir. Res..

[48] Lenz O., Verbinnen T., Lin T.I., Vijgen L., Cummings M.D., Lindberg J., Berke J.M., Dehertogh P., Fransen E., Scholliers A. (2010). In vitro resistance profile of the hepatitis C virus NS3/4A protease inhibitor TMC435. Antimicrob. Agents Chemother..

[49] Jiang M., Mani N., Lin C., Ardzinski A., Nelson M., Reagan D., Bartels D., Zhou Y., Nicolas O., Rao B.G. (2013). In vitro phenotypic characterization of hepatitis C virus NS3 protease variants observed in clinical studies of telaprevir. Antimicrob. Agents Chemother..

[50] Lin C., Lin K., Luong Y.P., Rao B.G., Wei Y.Y., Brennan D.L., Fulghum J.R., Hsiao H.M., Ma S., Maxwell J.P. (2004). In vitro resistance studies of hepatitis C virus serine protease inhibitors, VX-950 and BILN 2061: Structural analysis indicates different resistance mechanisms. J. Biol. Chem..

[51] Kieffer T.L., De Meyer S., Bartels D.J., Sullivan J.C., Zhang E.Z., Tigges A., Dierynck I., Spanks J., Dorrian J., Jiang M. (2012). Hepatitis C viral evolution in genotype 1 treatment-naïve and treatment-experienced patients receiving telaprevir-based therapy in clinical trials. PLoS ONE.

[52] Walker A., Filke S., Lübke N., Obermeier M., Kaiser R., Häussinger D., Timm J., Bock H.H. (2017). Detection of a genetic footprint of the sofosbuvir resistance-associated substitution S282T after HCV treatment failure. Virol. J..

[53] Group A.A.H., Vallet S., Viron F., Henquell C., Le Guillou-Guillemette H., Lagathu G., Abravanel F., Trimoulet P., Soussan P., Schvoerer E. (2011). NS3 protease polymorphism and natural resistance to protease inhibitors in French patients infected with HCV genotypes 1–5. Antivir. Ther..

[54] Morsica G., Andolina A., Merli M., Messina E., Hasson H., Lazzarin A., Uberti-Foppa C., Bagaglio S. (2017). NS3 protease resistance-associated substitutions in liver tissue and plasma samples from patients infected by hepatitis C virus genotype 1A or 1B. Arch. Virol..

[55] Aguiar B.F., Campos G.R.F., Rodrigues J.P.V., Marques N.N., Molina B.F., Bittar C., Souza F.F., Martinelli A.L.C., Rahal P., Pereira L.R.L. (2020). Baseline resistance associated substitutions in HCV genotype 1 infected cohort treated with Simeprevir, Daclatasvir and Sofosbuvir in Brazil. Clin. Res. Hepatol. Gastroenterol..

[56] Costa V.D., Delvaux N., Brandão-Mello C.E., Nunes E.P., de Sousa P.S.F., de Souza Rodrigues L., Lampe E., do Amaral Mello F.C. (2019). Prevalence of baseline NS3 resistance-associated substitutions (RASs) on treatment with protease inhibitors in patients infected with HCV genotype 1. Clin. Res. Hepatol. Gastroenterol..

[57] Dietz J., Müllhaupt B., Buggisch P., Graf C., Peiffer K.H., Matschenz K., Schattenberg J.M., Antoni C., Mauss S., Niederau C. (2023). Long-term persistence of HCV resistance-associated substitutions after DAA treatment failure. J. Hepatol..

[58] Itakura J., Kurosaki M., Kakizaki S., Amano K., Nakayama N., Inoue J., Endo T., Marusawa H., Hasebe C., Joko K. (2020). Features of resistance-associated substitutions after failure of multiple direct-acting antiviral regimens for hepatitis C. JHEP Rep..

[59] Gao M., Nettles R.E., Belema M., Snyder L.B., Nguyen V.N., Fridell R.A., Serrano-Wu M.H., Langley D.R., Sun J.H., O’Boyle D.R. (2010). Chemical genetics strategy identifies an HCV NS5A inhibitor with a potent clinical effect. Nature.

[60] Fridell R.A., Qiu D., Wang C., Valera L., Gao M. (2010). Resistance analysis of the hepatitis C virus NS5A inhibitor BMS-790052 in an in vitro replicon system. Antimicrob. Agents Chemother..

[61] Meanwell N.A., Belema M. (2019). The discovery and development of daclatasvir: An inhibitor of the hepatitis C virus NS5A replication complex. HCV: The Journey from Discovery to a Cure: Volume II.

[62] Pol S., Corouge M., Vallet-Pichard A. (2016). Daclatasvir–sofosbuvir combination therapy with or without ribavirin for hepatitis C virus infection: From the clinical trials to real life. Hepatic Med. Evid. Res..

[63] Stedman C. (2014). Sofosbuvir, a NS5B polymerase inhibitor in the treatment of hepatitis C: A review of its clinical potential. Ther. Adv. Gastroenterol..

[64] Flint M., Mullen S., Deatly A.M., Chen W., Miller L.Z., Ralston R., Broom C., Emini E.A., Howe A.Y. (2009). Selection and characterization of hepatitis C virus replicons dually resistant to the polymerase and protease inhibitors HCV-796 and boceprevir (SCH 503034). Antimicrob. Agents Chemother..

[65] Soumana D.I., Ali A., Schiffer C.A. (2014). Structural analysis of asunaprevir resistance in HCV NS3/4A protease. ACS Chem. Biol..

[66] McPhee F., Friborg J., Levine S., Chen C., Falk P., Yu F., Hernandez D., Lee M.S., Chaniewski S., Sheaffer A.K. (2012). Resistance analysis of the hepatitis C virus NS3 protease inhibitor asunaprevir. Antimicrob. Agents Chemother..

[67] Welsch C., Domingues F.S., Susser S., Antes I., Hartmann C., Mayr G., Schlicker A., Sarrazin C., Albrecht M., Zeuzem S. (2008). Molecular basis of telaprevir resistance due to V36 and T54 mutations in the NS3-4A protease of the hepatitis C virus. Genome Biol..

[68] Wyles D.L., Luetkemeyer A.F. (2017). Understanding Hepatitis C Virus Drug Resistance: Clinical Implications for Current and Future Regimens. Top. Antivir. Med..

[69] Tong X., Chase R., Skelton A., Chen T., Wright-Minogue J., Malcolm B.A. (2006). Identification and analysis of fitness of resistance mutations against the HCV protease inhibitor SCH 503034. Antivir. Res..

